# Lack of association between hypothyroxinemia of prematurity and transient thyroid abnormalities with adverse long term neurodevelopmental outcome in very low birth weight infants

**DOI:** 10.1371/journal.pone.0222018

**Published:** 2019-09-12

**Authors:** Lay Ong Tan, Mary Grace Tan, Woei Bing Poon

**Affiliations:** 1 Department of Paediatrics, KK Women's and Children's Hospital, Singapore, Singapore; 2 Department of Neonatal & Developmental Medicine, Singapore General Hospital, Singapore, Singapore; Metrohealth Medical Center, UNITED STATES

## Abstract

**Introduction:**

The association between hypothyroxinemia of prematurity with neurodevelopment was controversial.

**Objectives:**

To compare 5 year neurodevelopmental outcomes of very low birth weight (VLBW) infants with hypothyroxinemia of prematurity against those without.

**Methods:**

Retrospective cohort study in a single tertiary neonatal centre of VLBW infants born between the year 2008 to 2011. Comparisons were made between all abnormal and normal thyroid function controls using cord thyroid function tests, thyroid function tests during admission and pre-discharge thyroid function test done at term equivalent age. At 2 years corrected age, Bayley scales of infant and toddler development–third edition and Vineland II adaptive behaviour scales (VABS) were collected. At 5 years, Wechsler Preschool and Primary Scale of Intelligence (WPPSI-III), Bracken School Readiness Assessment, VABS and Beery Test of Visual-Motor Integration were collected.

**Results:**

110 subjects were studied at 2 years corrected age and 80 subjects at 5 years old. 29 infants had abnormal thyroid function test (10 infants with hypothyroxinemia of prematurity and 19 infants with transient thyroid abnormalities). There were no significant difference in the 2 years and 5 years developmental outcome between infants with and without hypothyroxinemia of prematurity (p-value>0.05); and between infants with and without transient thyroid abnormalities (p-value>0.05). There were no significant difference in neurological, visual and hearing impairment between infants with or without hypothyroxinemia of prematurity (p-value>0.05).

**Conclusions:**

Hypothyroxinemia of prematurity or transient thyroid abnormalities in VLBW infants were not associated with poorer neurodevelopment and did not support the need for levothyroxine supplementation.

## Introduction

Serum thyroxine (T4) and free T4 levels (fT4) alter in relation to the gestation in preterm infants in the first days after birth.[1] The concentrations of T4 and fT4 decrease to reach a nadir between day ten and fourteenth after birth that is more severe at lower gestations and birth weight.[1]

Transient hypothyroxinemia of prematurity (THOP) is defined as low level of circulating thyroid hormones, despite normal thyroid stimulating hormone (TSH) level.[2] THOP usually resolves within 2 to 3 weeks with progressive maturation of the hypothalamic-pituitary-thyroid axis.[3, 4]

Prevalence rates of THOP vary by study definition, affecting 35% [5] to 85% [6] of very preterm infants. Thyroid hormone is important for cerebral neurogenesis during early prenatal life [7]; neural migration and differentiation, axonal and dendritic growth, synaptogenesis [8, 9]; and gliogenesis.[2]

The relationship between THOP and poor neurodevelopmental outcomes was controversial. Delayed development of motor, cognitive, language, and educational skills were elicited in some studies.[10–13] However, prospective study by Scratch SE et al demonstrated that low fT4 levels in very preterm infants were not associated with adverse neuropsychological childhood outcomes.[2]

Transient hyperthyrotropinemia with elevated TSH is also common in preterm infants.[14] Initial hyperthyrotropinemia can bring about persistent hyperthyrotropinemia or permanent hypothyroidism requiring thyroid hormone replacement, or transient hypothyroidism that is benign in nature.

Further studies are needed to determine whether transient hypothyroxinemia of prematurity affects neurodevelopmental outcome.

## Objective

Our primary outcome is to compare 2 and 5 years neurodevelopmental outcomes of very low birth weight infants (VLBWIs) with hypothyroxinemia of prematurity against those without.

Our secondary outcome is to compare 2 and 5 years neurodevelopmental outcome of VLBWIs with transient thyroid abnormalities against those without.

## Methods

### Study area and population

This was a retrospective cohort study in a single tertiary neonatal centre of VLBWIs, defined as those with birthweights < 1500 grams, admitted between the years 2008 to 2011. Thyroid function tests were routinely performed in all infants from the cord blood at birth. Where these are insufficient or abnormal, repeat thyroid function tests were done at 1 week of life, and subsequently at 2 to 4 weekly intervals until normal range is achieved. In addition, a repeat thyroid function test is done at around term equivalent age, just before the baby was discharged.

Comparisons were made between subjects with any abnormal thyroid function tests and those with normal thyroid function controls. Patients who were diagnosed with congenital hypothyroidism were excluded.

### Laboratory confirmation of thyroid dysfunction

Reference ranges for TSH and fT4 was inferred from local Singapore population and based on thyroid hormone reference ranges published by Williams et al.[15] Normal cord TSH range is from 2.10mIU/L to 16.8mIU/L. Normal TSH range from day 5 of life to 1 month of life is between 1.23mIU/L to 11.5mIU/L, whereas normal TSH range from 31 days of life to 5 years of age is between 0.271 to 7.71mIU/L. Normal cord fT4 range is from 9.9pmol/L to 19.4pmol/L. Normal fT4 range from day 5 of life to 1 month of life is between 11.4pmol/L to 29.4pmol/L, whereas normal fT4 range from 31 days of life to 5 years of age is between 8.5pmol/L to 20.4pmol/L.

### Disease definition

Cases of THOP were defined based on International Classification of Diseases, Tenth Edition (ICD10) discharge diagnosis. Transient thyroid abnormalities was defined as isolated deranged TSH (raised or low) or isolated deranged fT4 (raised) which resolved on subsequent thyroid function test done before hospital discharge. These were grouped under other transient thyroid abnormalities.

Demographic factors comprised of gestational age, birth weight, gender, Apgar score at 1 and 5 minutes, antenatal chorioamnionitis and antenatal corticosteroid therapy. Gestational age was determined by the results of prenatal dating ultrasonography performed on or before 12 weeks of life and if dating ultrasound was not available, postnatal physical assessment was done using New Ballard score.[16] Morbidity variables included patent ductus arteriosus (PDA), sepsis, chronic lung disease (CLD), intraventricular hemorrhage (IVH), necrotizing enterocolitis (NEC), retinopathy of prematurity (ROP) [17] and postnatal steroids therapy. Sepsis was defined as those with a positive blood culture or infants with clinical signs of systemic infection. CLD was characterised by oxygen dependency for the first 28 days of life.[18] The presence of IVH was determined by ultrasonographic scanning of the brain based on Volpe classification.[19] Periventricular leukomalacia (PVL) was characterised by cranial ultrasound finding of initial periventricular echodensities, followed later by cystic formation. NEC was classified based on Bell’s criteria (at least stage 1 Bell’s criteria).[20]

Cerebral palsy was defined based on neurologic findings by the attending doctor, with at least one of the following criteria: the inability to walk five steps unaided by the age of two years; motor disability requiring physical therapy; motor disorder requiring surgical intervention; or the use of braces or other physical-assistance devices.

### Outcome measures

Children were assessed as close as possible to 24 months corrected age and 5 years of age by paediatricians and clinical psychologists. Assessments included clinical and neurologic examinations, visual assessment, hearing assessment, and developmental assessments using the Bayley Scales of Infant and Toddler Development, Third Edition (Bayley-III) [21, 22], and Vineland II adaptive behaviour scales (VABS) at 2 years old; and the Wechsler Preschool and Primary Scale of Intelligence (WPPSI) test, Bracken School Readiness Assessment, VABS and Beery Test of Visual-Motor Integration in children aged 5 years.[23]

Neurodevelopmental impairment (NDI) was considered if the patient had any of the following: (1) Bayley-III assessment score ≥1 SD below the sample mean in any of the subscales (cognitive, language, or motor) [22]; (2) WPSSI full scale composite IQ score >1 SD below the population mean [23]; (3) cerebral palsy at a Gross Motor Function Classification System score ≥2; (4) any abnormality on neurological examination; (5) any hearing impairment; or (6) bilateral blindness. Hearing loss with or without a hearing aid was considered abnormal, but correctable visual impairment such as myopia was considered normal.

Primary clinical outcome measures were neurodevelopmental status at 2 years and 5 years follow up. The developmental assessment tool (summarised in Table 1) was administrated by trained and accredited psychologists.

**Table 1:** Neuropsychological measures.

Test battery	Description
WPPSI-III	To measure the cognitive ability of children aged 2 years 6 months through 7 years 3 months. It provides a composite score that represents general intellectual ability (Full Scale IQ score) that is derived from seven core subtests. Normative mean = 100, SD = 15.
Bayley-III	Assesses development of children between the ages of 1 month and 42 months. Cognitive development, expressive and receptive language, and fine and gross motor development are all evaluated. Composite scores are derived for cognitive, language, and motor development and scaled to a metric, with a mean of 100, standard deviation of 15, and range of 40 to 160.
VABS	Vineland Adaptive Behaviour Scales, Second Edition (Vineland-II) is a questionnaire that measures general adaptive behaviour, which refers to the ability to carry out day-to-day activities necessary to take care of oneself and get along with others. (normative mean = 100, standard deviation = 15).
Bracken	The Bracken School-Readiness Assessment evaluates the understanding of foundational concepts in the categories of colours, letters, numbers, sizing, comparisons, and shapes. The standardised scores have a normative mean of 100 and a standard deviation of 15.
Beery	The Beery Test of visual-motor integration (VMI) assesses the ability to copy geometric forms. Raw scores were converted to standard scores (mean standard score: 100; SD: 15).

The Bayley Scales of Infant and Toddler Development, third edition (Bayley III), was used to assess development of children between the ages of 1 month and 42 months.[22] In the Bayley III, cognitive development, expressive and receptive language, and fine and gross motor development were all evaluated. Composite scores were derived for cognitive, language, and motor development and scaled to a metric, with a mean of 100, standard deviation of 15, and range of 40 to 160. Results were also expressed as percentile ranks relative to the standardization sample, with a mean and median of 50 and range from 1 to 99.

The WPPSI-III (2002) was an individually administered intelligence test.[24] Full Scale IQ (FSIQ) (normative mean = 100 and standard deviation = 15) was derived from seven core subtests that provided Verbal, Performance, and Processing Speed scores. It was usually considered the most representative measure of global intellectual functioning. The Verbal IQ (VIQ) was a measure of acquired knowledge, verbal reasoning, and comprehension of verbal information. The Performance IQ (PIQ) is a measure of a child’s nonverbal reasoning, spatial processing skills, attentiveness to detail, and visual–motor coordination skills. The Processing Speed Quotient (PSQ) provides a measure of child’s ability to quickly and correctly scan, sequence, and discriminate simple visual information.

Vineland Adaptive Behaviour Scales, Second Edition (Vineland-II) was administered as a questionnaire that measured general adaptive behaviour, which was the ability to carry out day-to-day activities necessary to take care of oneself and get along with others.[25] Parents were asked to fill in the Vineland-II Parent/Caregiver Rating Form. Adaptive behaviour was defined as performance of day-to-day activities necessary to take care of oneself and get along with others. The examiner then checked through the form to ensure that both baselines and ceilings were met. The Vineland-II was designed to be administered individually (normative mean = 100, standard deviation = 15). Eleven general subdomains were grouped into four domains: communication, daily living skills, socialization, and motor skills. The domains were made up of subdomains in which the scores were added to form the domain composite scores. The four domain composite scores were then combined to form the adaptive behaviour composite for those individuals aged birth to 6 years 11 months.

The Bracken School-Readiness Assessment evaluated understanding of foundational concepts in the categories of colours, letters, numbers, sizing, comparisons, and shapes to assess for school readiness.[26] Raw scores were provided for each individual subtest, and were added together to provide a composite raw score, known as the School Readiness Composite. The composite raw score was used to produce an age-adjusted standardised composite score. The standardised scores had a mean of 100 and a standard deviation of 15.

The Beery Test of visual-motor integration (VMI) assessed the ability to copy geometric forms. Raw scores were converted to standard scores (mean standard score: 100; SD: 15).[27]

Need for a hearing aid made up the criterion for hearing impairment. Children with corrected visual acuity between 20/60 and 20/200 were defined as visually impaired, and children with visual acuity worse than 20/200 were treated as blind.

### Data analysis and ethics approval

Data was retrieved through a review of medical records of eligible patients. Laboratory, microbiological, radiological, treatment and outcome data were extracted.

Data were recorded and analysed using statistical software (SPSS version 20; SPSS Inc., Chicago, IL, USA). The categorical variables were compared by chi-square test. Normally distributed continuous data were compared by one way ANOVA test, and data not conforming to normal distribution were compared using the independent samples Kruskall-Wallis test. P values were considered statistically significant at <0.05.

The study was approved by the Singhealth Centralised Institutional Review Board, and granted waiver of consent.

## Results

**Fig 1:**
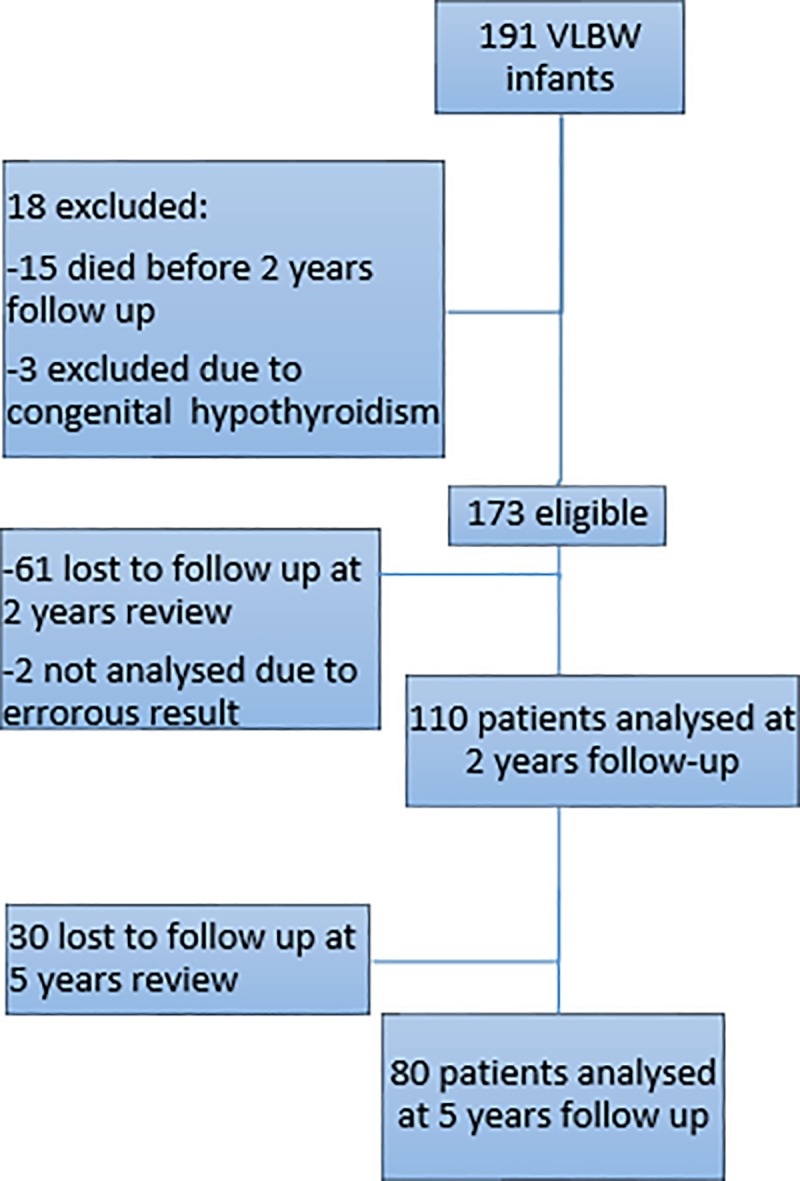
Flow chart of the study population.

There were 191 VLBWIs during the study period, with a total of 110 patients and 80 patients analysed at 2 years and 5 years follow-up respectively. 29 patients out of 110 patients at 2 years follow up, and 22 patients out of 80 patients at 5 years follow up were not administered developmental assessment tool by trained and accredited psychologists as parents opt out of the assessment during the follow up. (Fig 1) Their baseline characteristics were described in Table 2.

**Table 2:** Baseline characteristics of the study population.

Thyroid status	Hypothyroxinemia of prematurity (N = 10)	Other transient thyroid abnormalities (N = 19)	Normal thyroid function test (N = 81)	
Characteristics	No. (%), unless otherwise stated	No. (%), unless otherwise stated	No. (%), unless otherwise stated	p-value
Gender, male; n (%)$	4 (40)	9 (47.4)	45 (55.6)	0.569
Gestational age at birth (weeks); mean (SD)*	26.4 ± 1.9	29.6 ± 2.2	28.9 ± 2.2	0.001
Birth weight (gram); mean (SD)*	801 (179)	1053 (297)	1057 (249)	0.012
Race; n (%)$				
Malay	1 (10)	4 (21.1)	15 (18.5)	0.989
Chinese	7 (70)	11 (57.9)	50 (61.7)	
Indian	1 (10)	2 (10.5)	6 (7.4)	
Others	1(10)	2 (10.5)	10 (12.3)	
Apgar score, median (IQR)@				
1 minute	5 (5, 6)	7 (6. 8)	7 (5, 8)	0.073
5 minutes	8 (7, 8)	9 (8, 9)	9 (8, 9)	0.053
Antenatal steroids; n (%)$	9 (90)	16 (84.2)	75 (93.8)	0.389
Maternal chorioamnionitis; n (%)$	1 (10)	2 (10.5)	16 (19.8)	0.516
Singleton birth; n (%)$	6 (60)	14 (73.7)	63 (77.8)	0.459
Intraventricular haemorrhage; n (%)$	1 (10)	2 (10.5)	8 (9.9)	0.996
Periventricular leukomalacia; n (%)$	0 (0)	0 (0)	1 (1.2)	0.835
Patent ductus arteriosus; n (%)$	6 (60)	11 (57.9)	32 (39.5)	0.205
Chronic lung disease; n (%)$	7 (70)	2 (10.5)	31 (38.3)	0.005
Postnatal steroids; n (%)$	3 (42.9)	0 (0)	4 (12.9)	0.136
Necrotising enterocolitis; n (%)$	0 (0)	1 (5.3)	7 (8.6)	0.57
Retinopathy of prematurity; n (%)$	5 (50)	3 (17.6)	20 (24.7)	0.158
Sepsis; n (%)$	8 (80)	8 (42.1)	46 (56.8)	0.146

IQR: interquartile range

$ chi-square

* one way ANOVA, SD: standard deviation

@ Kruskal-Wallis test

The mean gestational age of infants with hypothyroxinaemia of prematurity (THOP) was significantly lower at 26.4 weeks (p = 0.001) as compared with those without. The mean birth weight was lower in infants with THOP (801 grams, p = 0.012) as compared with those without. Infants with THOP had experienced CLD more than infants with normal thyroid function test (p = 0.005). Otherwise, the different thyroid status groups were comparable in regards to other comorbidities (including IVH, PVL, PDA, CLD, ROP, sepsis and NEC).

Table 3 summarised the outcomes at 2 years old. There were no increase in cerebral palsy, epilepsy, visual or hearing impairment in patients with THOP as compared to those without (p>0.55). Using Bayley-III assessment, the cognitive, language and motor score were similar across all thyroid status groups (p>0.2). Patients with THOP had similar adaptive behavioural score (Vineland) as compared to those without.

**Table 3:** Neurodevelopmental outcome at 2 years old.

Neurodevelopmental Follow up at 2 years old	Hypothyroxinemia of prematurity (N = 10)	Other transient thyroid abnormalities (N = 19)	Normal thyroid function test (N = 81)	p-value
Chronological age at follow up (months); mean (SD) *	25.2 (2)	25.8 (2.5)	25 (2.3)	0.357
Corrected age at follow up (months); mean (SD)*	20.3 (0.6)	23.7 (3.3)	22.3 (2.9)	0.214
Cerebral palsy (GMFCS >2), n (%)$	0 (0)	1 (5.3)	3 (3.7)	0.77
Epilepsy, n (%)$	0 (0)	0 (0)	2 (2.5)	0.694
Visual impairment, n (%)$	1 (10)	1 (5.3)	6 (7.4)	0.893
Hearing impairment (any), n (%)$	0 (0)	0(0)	3 (3.7)	0.576
Bayley-III score <85 at 2 y corrected age, n (%)$ (n = 81)	(n = 8)	(n = 12)	(n = 61)	
Cognitive score <85	1 (14.3)	4 (33.3)	9 (14.8)	0.293
Language score <85	3 (42.9)	4 (40)	22 (38.6)	0.975
Motor score <85	2 (25)	5 (41.7)	11 (19.6)	0.264
Bayley-III score at 2 y corrected age, mean (SD)* (n = 81)	(n = 8)	(n = 12)	(n = 61)	
Cognitive	93.6 (12.8)	92.5 (19)	96.4 (13.6)	0.646
Language	82.9 (19.1)	84.6 (13.9)	86 (16.1)	0.875
Motor	92.1 (12)	89.1 (15.3)	93.3 (13.8)	0.629
Vineland-II Adaptive Behaviour Scales, mean (SD) * (n = 81)	(n = 9)	(n = 12)	(n = 60)	
Adaptive behaviour composite score	94 (15.1)	89.8 (12.7)	93.1 (11.7)	0.662
Communication score	92.8 (13.9)	87.1 (14.3)	93.8 (12.2)	0.25
Daily living skills score	98.1 (14.5)	92.6 (13.4)	95.4 (14.3)	0.683
Socialisation score	90.9 (13.7)	88.3 (8.5)	91.6 (10.2)	0.593
Motor skills score	99 (13)	95.6 (10.1)	98.2 (10.5)	0.708

$ chi-square

* one way ANOVA

SD: standard deviation, GMFCS: Gross Motor Function Classification System.

Similarly at 5 years follow-up, there were no increase in significant impairment in patients with THOP as compared to those without (p = 0.787) (Table 4). Using WPPSI-III assessment, the full scale composite IQ score, verbal IQ score, performance IQ score and processing speed quotient score were similar across all thyroid status groups (p>0.1). Patients with THOP had similar adaptive behavioural score (Vineland) as compared to those without (p = 0.311). Infants with THOP also had similar school readiness composite standard score (Bracken, p = 0.304) and Beery standard score (p = 0.833) as compared to other thyroid status groups.

**Table 4:** Neurodevelopmental outcome at 5 years old.

Neurodevelopmental Follow up at 5 years old	Hypothyroxinemia of prematurity (N = 10)	Other transient thyroid abnormalities (N = 19)	Normal thyroid function test (N = 81)	p-value
Chronological age at follow up (months); mean (SD)*	61.9 (3.5)	63.4 (5)	61.9 (8.8)	0.827
WPPSI-III score <85, n (%)$ (n = 58)	(n = 8)	(n = 7)	(n = 43)	
Full scale composite IQ score	1 (16.7)	2 (40)	5 (13.9)	0.346
Verbal IQ score	3 (37.5)	1 (14.3)	15 (34.9)	0.534
Performance IQ score	1 (14.3)	2 (28.6)	5 (11.6)	0.489
Processing speed quotient score	1 (14.3)	1 (16.7)	4 (12.5)	0.96
WPPSI-III, mean (SD)* (n = 58)	(n = 8)	(n = 7)	(n = 43)	
Full scale composite IQ score	97.8 (17.8)	90.4 (18.5)	93.5 (14.4)	0.714
Verbal IQ score	86.9 (12)	90.5 (10.2)	87.1 (12)	0.762
Performance IQ score	106.1 (20.6)	104.3 (25.6)	102.3 (16.1)	0.856
Processing speed quotient score	108.5 (15.1)	91.8 (11.8)	104.8 (15.4)	0.109
Vineland-II Adaptive Behaviour Scales, mean (SD)* (n = 6)	(n = 2)	(n = 0)	(n = 4)	
Adaptive behaviour composite score	70 (-)	-	66 (2.9)	0.311
Communication score	79.5 (17.7)	-	70 (2.4)	0.294
Daily living skills score	88.5 (16.3)	-	67 (6.7)	0.068
Socialisation score	85.5 (12)	-	67 (6.7)	0.081
Motor skills score	88.5 (19.1)	-	72.3 (6)	0.16
Bracken school readiness assessment 3rd edition, mean (SD)* (n = 55)	(n = 8)	(n = 7)	(n = 40)	
School readiness composite standard score	87.8 (18.7)	82.4 (24.5)	92.9 (15.8)	0.304
Beery VMI 5th edition, mean (SD)* (n = 50)	(n = 7)	(n = 5)	(n = 38)	
Beery standard score	107 (6.1)	104.8 (9.9)	107.4 (9.3)	0.833
Significant impairment at 5 years old, n (%)$	6 (60)	9 (47.4)	44 (54.3)	0.787

$ chi-square

* one way ANOVA

SD: standard deviation, WPPSI-III: Wechsler Preschool and primary scale of intelligence 3rd edition. GMFCS: Gross Motor Function Classification System. Significant impairment is defined as any composite Bayley-III score <85, WPPSI-III full scale composite IQ score <85, any hearing loss, blindness, or cerebral palsy with GCFMS score >2. Includes Bayley-III, n = 81; WIPPSI, n = 58.

## Discussion

Gestational age and birthweight were important risk factors for poorer neurodevelopmental outcomes in VLBWIs, although other morbidities such as IVH, CLD, ROP and NEC were also associated with poorer neurodevelopmental outcomes.[28–31] In our study, patients with THOP had lower gestational age, lower birth weight and higher incidence of CLD, but there was no significant difference in the neurodevelopmental outcome as compared with patients without THOP.

The risk factors [32] for transient hypothyroxinaemia in preterm infants included lower gestational age [1, 6, 33–36], maternal preeclampsia with placental insufficiency [37], fetal growth restriction [5], perinatal asphyxia [38], respiratory distress syndrome (RDS) [5, 33], more severe respiratory disease [34], mechanical ventilation [34, 35], low diastolic blood pressure [34] and dopamine infusions [35, 36]. Adverse neonatal outcomes were associated with transient hypothyroxinaemia, including intraventricular haemorrhage [6, 39], chronic lung disease [34] and death.[6, 34, 40] The finding of association between chronic lung disease and THOP was also observed in our study.

Van Wassenaer et. al. revealed that low thyroid hormone levels was associated with neurodevelopmental impairments in preterm infants.[41] They had previously reported that low fT4 levels in the first postnatal month was associated with poorer neurodevelopmental outcome in premature infants with a gestational age less than 30 weeks.[42]

In our study, we did not find association between THOP or transient thyroid abnormalities with adverse neurodevelopmental outcomes at 2 or 5 years. We postulated that for those studies in the literature which found associations between thyroid abnormalities in VLBWIs and adverse neurodevelopmental outcomes, the thyroid status may be merely a confounder, namely that they reflected the severity of illness or of comorbities such as intraventricular haemorrhage, and these were the real risk factors associated with neurodevelopmental impairment. Our postulation was supported by studies from Fisher et al which found that the low thyroxine concentrations and low triiodothyronine concentrations typically seen in preterm infants were the result of non-thyroidal illness and reflected the severity of illness in preterm infants.[43] In such instances, thyroid hormone production falls and protein catabolism and oxygen consumption may be reduced—a potentially beneficial adaptive response to illness. In addition, studies by Williams et al on the impact of RDS, IVH, NEC and sepsis on thyroid function [44] and on PDA and hypotensive preterm infants on inotropes by Carrascossa et al. further supported this phenomenon.[45]

A Cochrane review published by Osborn DA et al did not back the use of prophylactic thyroid hormones in preterm infants to reduce neonatal mortality, morbidity or ameliorate neurodevelopmental outcomes.[32, 46] Furthermore, it was uncertain whether thyroid hormone treatment for THOP could enhance neurodevelopmental outcome in preterm infants. On the contrary, delayed replacement of thyroid hormone in infants with congenital hypothyroidism may cause adverse neurodevelopmental outcome. Going by the results of our study, which did not show any difference in neurodevelopmental outcomes in those with THOP or transient thyroid abnormalities, this did not seem to support the need for thyroxine replacement in these groups of VLBWIs. Hypothyroxinemia caused by non-thyroidal illness reflects the severity of the underlying illness and this could be protective, therefore thyroid hormone supplementation might not be appropriate.[47]

The strength of our study was that there was a systematic and standardised follow-up protocol. Infants with THOP were not treated with thyroid hormone supplement in the study, which allowed meaningful comparisons of outcomes with the controls. Although there was a difference of 3 weeks between the THOP group and the controls, our assessment at 2 years was using corrected age, which essentially accounted for the difference between the groups and found no significant differences in neurodevelopmental outcomes, and there were no significant differences in other co-morbidities except chronic lung disease. Similarly at 5 years, no difference was found in neurodevelopmental outcomes.

Our study had limitations because of its retrospective nature and was a single centre analysis with relatively lesser numbers, particularly by the 5 years follow-up.

## Conclusion

Hypothyroxinemia of prematurity or transient thyroid abnormalities in VLBWIs were not associated with poorer neurodevelopment and did not support the need for supplementation.

## References

[1] Rooman RP, Du Caju MV, De Beeck LO, Docx M, Van Reempts P, Van Acker KJ. Low thyroxinaemia occurs in the majority of very preterm newborns. Eur J Pediatr. 1996;155(3):211–5. . 8929730 10.1007/BF01953940

[2] Scratch SE, Hunt RW, Thompson DK, Ahmadzai ZM, Doyle LW, Inder TE, et al Free thyroxine levels after very preterm birth and neurodevelopmental outcomes at age 7 years. Pediatrics. 2014;133(4):e955–63. 10.1542/peds.2013-2425 24685955 PMC3966502

[3] Mercado M, Yu VY, Francis I, Szymonowicz W, Gold H. Thyroid function in very preterm infants. Early Hum Dev. 1988;16(2–3):131–41. . 3378519 10.1016/0378-3782(88)90093-x

[4] van Wassenaer AG, Kok JH, Dekker FW, de Vijlder JJ. Thyroid function in very preterm infants: influences of gestational age and disease. Pediatr Res. 1997;42(5):604–9. 10.1203/00006450-199711000-00009 . 9357931

[5] Uhrmann S, Marks KH, Maisels MJ, Kulin HE, Kaplan M, Utiger R. Frequency of transient hypothyroxinaemia in low birthweight infants. Potential pitfall for neonatal screening programmes. Arch Dis Child. 1981;56(3):214–7. 10.1136/adc.56.3.214 7212760 PMC1627144

[6] Paul DA, Leef KH, Stefano JL, Bartoshesky L. Low serum thyroxine on initial newborn screening is associated with intraventricular hemorrhage and death in very low birth weight infants. Pediatrics. 1998;101(5):903–7. 10.1542/peds.101.5.903 . 9565423

[7] Ares S, Quero J, Escobar-Morreale H. Thyroid hormone metabolism in premature infants and their neurodevelopment The Thyroid and Brain Stuttgart, Germany: Schattauer GmbH 2003:85–96.

[8] Balazs R, Kovacs S, Cocks WA, Johnson AL, Eayrs JT. Effect of thyroid hormone on the biochemical maturation of rat brain: postnatal cell formation. Brain Res. 1971;25(3):555–70. . 5544324 10.1016/0006-8993(71)90460-4

[9] Khamsi F, Eayrs JT. A study of the effects of thyroid hormones on growth and development. Growth. 1966;30(2):143–56. . 5963694

[10] Delahunty C, Falconer S, Hume R, Jackson L, Midgley P, Mirfield M, et al Levels of neonatal thyroid hormone in preterm infants and neurodevelopmental outcome at 5½ years: Millennium cohort study. The Journal of Clinical Endocrinology & Metabolism. 2010;95(11):4898–908. 20719832 10.1210/jc.2010-0743

[11] Reuss ML, Paneth N, Pinto-Martin JA, Lorenz JM, Susser M. The relation of transient hypothyroxinemia in preterm infants to neurologic development at two years of age. N Engl J Med. 1996;334(13):821–7. 10.1056/NEJM199603283341303 . 8596548

[12] Den Ouden A, Kok J, Verkerk P, Brand R, Verloove-Vanhorick S. The relation between neonatal thyroxine levels and neurodevelopmental outcome at age 5 and 9 years in a national cohort of very preterm and/or very low birth weight infants. Pediatric research. 1996;39(1):142 8825399 10.1203/00006450-199601000-00021

[13] Meijer WJ, Verloove-Vanhorick SP, Brand R, van den Brande JL. Transient hypothyroxinaemia associated with developmental delay in very preterm infants. Arch Dis Child. 1992;67(7):944–7. 10.1136/adc.67.7.944 1381573 PMC1793825

[14] Lee JH, Kim SW, Jeon GW, Sin JB. Thyroid dysfunction in very low birth weight preterm infants. Korean journal of pediatrics. 2015;58(6):224 10.3345/kjp.2015.58.6.224 26213551 PMC4510356

[15] Williams FL, Simpson J, Delahunty C, Ogston SA, Bongers-Schokking JJ, Murphy N, et al Developmental trends in cord and postpartum serum thyroid hormones in preterm infants. The Journal of Clinical Endocrinology & Metabolism. 2004;89(11):5314–20. 15531476 10.1210/jc.2004-0869

[16] Ballard JL, Khoury JC, Wedig K, Wang L, Eilers-Walsman BL, Lipp R. New Ballard Score, expanded to include extremely premature infants. J Pediatr. 1991;119(3):417–23. . 1880657 10.1016/s0022-3476(05)82056-6

[17] Prematurity ICftCoRo. The international classification of retinopathy of prematurity revisited. Archives of ophthalmology (Chicago, Ill: 1960). 2005;123(7):991. 10.1001/archopht.123.7.991 16009843

[18] Kinsella JP, Greenough A, Abman SH. Bronchopulmonary dysplasia. The Lancet. 2006;367(9520):1421–31. 10.1016/S0140-6736(06)68615-7 16650652

[19] Inder TE, Perlman JM, Volpe JJ. Preterm intraventricular hemorrhage/posthemorrhagic hydrocephalus. Volpe's neurology of the newborn: Elsevier; 2018 p. 637–98. e21.

[20] Kliegman R, Walsh M. Neonatal necrotizing enterocolitis: pathogenesis, classification, and spectrum of illness. Current problems in pediatrics. 1987;17(4):219–88. 10.1016/0045-9380(87)90031-4 PMC7130819 3556038

[21] Goh SK, Tham EK, Magiati I, Sim L, Sanmugam S, Qiu A, et al Analysis of item-level bias in the Bayley-III language subscales: the validity and utility of standardized language assessment in a multilingual setting. Journal of Speech, Language, and Hearing Research. 2017;60(9):2663–71. 10.1044/2017_JSLHR-L-16-0196 28813555

[22] Bayley N. Bayley scales of infant and toddler development: PsychCorp, Pearson; 2006.

[23] Bode MM, D'Eugenio DB, Mettelman BB, Gross SJ. Predictive validity of the Bayley, at 2 years for intelligence quotient at 4 years in preterm infants. Journal of Developmental & Behavioral Pediatrics. 2014;35(9):570–5. 25370298 10.1097/DBP.0000000000000110

[24] Lichtenberger EO, Kaufman AS. Essentials of WPPSI-III Assessment: John Wiley & Sons Inc; 2004.

[25] Sparrow SS, Balla DA, Cicchetti DV. Vineland-II: Survey Forms Manual; Vineland Adaptive Behavior Scales; Survey Interview Form and Parent/caregiver Rating Form; a Revision of the Vineland Social Maturity Scale by Edgar A. Doll: Pearson Assessments; 2005.

[26] Bracken BA. School Readiness Assessment: Examiner's manual: Pearson; 2007.

[27] Beery KE. Beery VMI: The Beery-Buktenica developmental test of visual-motor integration. Minneapolis, MN: Pearson 2004.

[28] Taylor GH, Klein NM, Minich NM, Hack M. Verbal memory deficits in children with less than 750 g birth weight. Child Neuropsychol. 2000;6(1):49–63. 10.1076/0929-7049(200003)6:1;1-B;FT049 . 10980668

[29] Bhutta AT, Cleves MA, Casey PH, Cradock MM, Anand KJ. Cognitive and behavioral outcomes of school-aged children who were born preterm: a meta-analysis. JAMA. 2002;288(6):728–37. . 12169077 10.1001/jama.288.6.728

[30] Anderson PJ, De Luca CR, Hutchinson E, Spencer-Smith MM, Roberts G, Doyle LW, et al Attention problems in a representative sample of extremely preterm/extremely low birth weight children. Dev Neuropsychol. 2011;36(1):57–73. 10.1080/87565641.2011.540538 . 21253991

[31] Barre N, Morgan A, Doyle LW, Anderson PJ. Language abilities in children who were very preterm and/or very low birth weight: a meta-analysis. J Pediatr. 2011;158(5):766–74 e1. 10.1016/j.jpeds.2010.10.032 . 21146182

[32] Osborn DA, Hunt R. Prophylactic postnatal thyroid hormones for prevention of morbidity and mortality in preterm infants. Cochrane Database of Systematic Reviews. 2007;(1). 10.1002/14651858.CD005948.pub2 PMC9004229 17253571

[33] Franklin R, Purdie G, O'Grady C. Neonatal thyroid function: prematurity, prenatal steroids, and respiratory distress syndrome. Archives of disease in childhood. 1986;61(6):589–92. 10.1136/adc.61.6.589 3729529 PMC1777818

[34] Reuss ML, Leviton A, Paneth N, Susser M. Thyroxine values from newborn screening of 919 infants born before 29 weeks' gestation. American journal of public health. 1997;87(10):1693–7. 9357357 10.2105/ajph.87.10.1693 PMC1381138

[35] Herring MJK, Leef KH, Locke RG, Stefano JL, Bartoshesky L, Paul DA. Are perinatal risk factors helpful in predicting and optimizing treatment strategies for transient hypothyroxinemia in very-low-birth-weight infants? American journal of perinatology. 2003;20(06):333–40. 14528403 10.1055/s-2003-42691

[36] Filippi L, Cecchi A, Tronchin M, Dani C, Pezzati M, Seminara S, et al Dopamine infusion and hypothyroxinaemia in very low birth weight preterm infants. European journal of pediatrics. 2004;163(1):7–13. 14648215 10.1007/s00431-003-1359-8

[37] Belet N, Imdat H, Yanık F, Küçüködük Ş. Thyroid function tests in preterm infants born to preeclamptic mothers with placental insufficiency. Journal of Pediatric Endocrinology and Metabolism. 2003;16(8):1131–6. 14594173 10.1515/jpem.2003.16.8.1131

[38] Tahirovic H. Transient Hypothyroxincmia in Neonates with Birth Asphyxia Delivered by Emergency Cesarean Section. De Gruyter; 1994. 10.1515/jpem.1994.7.1.39 8186822

[39] Paul DA, Leef KH, Stefano JL, Bartoshesky L. Thyroid function in very-low-birth-weight infants with intraventricular hemorrhage. Clinical pediatrics. 2000;39(11):651–6. 11110365 10.1177/000992280003901104

[40] Hsu C-H, Chang J, Lee Y-J, Hung H-Y, Kao H-A, Huang F-Y. Thyroid function in the sick very low-birth-weight infants. Acta paediatrica Taiwanica = Taiwan er ke yi xue hui za zhi. 1999;40(4):237–42. 10910620

[41] van Wassenaer AG, Kok JH, editors. Hypothyroxinaemia and thyroid function after preterm birth. Seminars in Neonatology; 2004: Elsevier. 10.1016/S1084-2756(03)00114-3 15013471

[42] van Wassenaer AG, Briet JM, van Baar A, Smit BJ, Tamminga P, de Vijlder J, et al Free thyroxine levels during the first weeks of life and neurodevelopmental outcome until the age of 5 years in very preterm infants. Pediatrics. 2002;110(3):534–9. 12205256 10.1542/peds.110.3.534

[43] Fisher DA. Euthyroid low thyroxine (T4) and triiodothyronine (T3) states in prematures and sick neonates. Pediatric clinics of North America. 1990;37(6):1297–312. 2259541 10.1016/s0031-3955(16)37012-2

[44] Williams FL, Ogston SA, van Toor H, Visser TJ, Hume R, Group wcftSPT. Serum thyroid hormones in preterm infants: associations with postnatal illnesses and drug usage. The Journal of Clinical Endocrinology & Metabolism. 2005;90(11):5954–63. 16105964 10.1210/jc.2005-1049

[45] Carrascosa A, Ruiz-Cuevas P, Clemente M, Salcedo S, Almar J. Thyroid function in 76 sick preterm infants 30–36 weeks: results from a longitudinal study. Journal of Pediatric Endocrinology and Metabolism. 2008;21(3):237–44. 18540250 10.1515/jpem.2008.21.3.237

[46] Osborn DA, Hunt R. Postnatal thyroid hormones for preterm infants with transient hypothyroxinaemia. Cochrane Database of Systematic Reviews. 2007;(1). 10.1002/14651858.CD005945.pub2 PMC7388860 17253568

[47] Hennemann G, Docter R, Krenning E. Causes and effects of the low T3 syndrome during caloric deprivation and non-thyroidal illness: an overview. Acta Medica Austriaca. 1988;15:42–5. 3051835

