# Correction: Lack of association between hypothyroxinemia of prematurity and transient thyroid abnormalities with adverse long term neurodevelopmental outcome in very low birth weight infants

**DOI:** 10.1371/journal.pone.0223867

**Published:** 2019-10-09

**Authors:** Lay Ong Tan, Mary Grace Tan, Woei Bing Poon

There are errors in the author affiliations. The correct affiliations are as follows:

Lay Ong Tan^1,2^, Mary Grace Tan^2^, Woei Bing Poon^2^

**1** Department of Paediatrics, KK Women's and Children's Hospital, Singapore, **2** Department of Neonatal & Developmental Medicine, Singapore General Hospital, Singapore.
